# Histamine from Brain Resident MAST Cells Promotes Wakefulness and Modulates Behavioral States

**DOI:** 10.1371/journal.pone.0078434

**Published:** 2013-10-18

**Authors:** Sachiko Chikahisa, Tohru Kodama, Atsushi Soya, Yohei Sagawa, Yuji Ishimaru, Hiroyoshi Séi, Seiji Nishino

**Affiliations:** 1 Sleep & Circadian Neurobiology Laboratory, Stanford University School of Medicine, Palo Alto, California, United States of America; 2 Department of Integrative Physiology, Institute of Health Biosciences, the University of Tokushima Graduate School, Tokushima, Japan; 3 Department of Psychophysiology, Tokyo Metropolitan Institute of Medical Science, Tokyo, Japan; Kent State University, United States of America

## Abstract

Mast cell activation and degranulation can result in the release of various chemical mediators, such as histamine and cytokines, which significantly affect sleep. Mast cells also exist in the central nervous system (CNS). Since up to 50% of histamine contents in the brain are from brain mast cells, mediators from brain mast cells may significantly influence sleep and other behaviors. In this study, we examined potential involvement of brain mast cells in sleep/wake regulations, focusing especially on the histaminergic system, using mast cell deficient (W/W^v^) mice. No significant difference was found in the basal amount of sleep/wake between W/W^v^ mice and their wild-type littermates (WT), although W/W^v^ mice showed increased EEG delta power and attenuated rebound response after sleep deprivation. Intracerebroventricular injection of compound 48/80, a histamine releaser from mast cells, significantly increased histamine levels in the ventricular region and enhanced wakefulness in WT mice, while it had no effect in W/W^v^ mice. Injection of H1 antagonists (triprolidine and mepyramine) significantly increased the amounts of slow-wave sleep in WT mice, but not in W/W^v^ mice. Most strikingly, the food-seeking behavior observed in WT mice during food deprivation was completely abolished in W/W^v^ mice. W/W^v^ mice also exhibited higher anxiety and depression levels compared to WT mice. Our findings suggest that histamine released from brain mast cells is wake-promoting, and emphasizes the physiological and pharmacological importance of brain mast cells in the regulation of sleep and fundamental neurobehavior.

## Introduction

Mast cells are derived from hematopoietic stem cells and complete their differentiation under local tissue microenvironmental factors when they enter tissues and organs  [1-3]. Mast cells are known for their role in allergic inflammation and in host defense to immunologic stimuli in peripheral tissues [1,4-6]. Mast cells also populate the brain of many mammalian species, including rodents and humans  [7,8]. Mast cells have been observed in various brain structures, including the brain side of the blood-brain barrier, thalamus, entorhinal cortex, hippocampus, and the leptomeninges overlying these areas [9-12]. Mast cells in the brain are active in the basal state and release their contents by piecemeal or anaphylactic degranulation [9,13]. They contain numerous mediators including classical neurotransmitters, cytokines, chemokines, and lipid-derived factors [7,8]. These mediators are secreted from mast cells upon receiving an appropriate signal and in turn influence neuronal activity of central nervous system (CNS) and vascular permeability.

Although the activity of brain mast cells is increased by multiple stimuli including nerve growth factor (NGF), corticotrophin releasing hormone (CRH), chatecholamines, and substance P [14], their physiological role remains unclear. In addition, the number of brain mast cells is highly affected by the behavioral state of the animal; chronic subordination stress such as exposure to a fighting opponent increased the number of brain mast cells in mice [15], while social stress of isolation markedly reduced the total number of brain mast cells [16]. Thus, many fundamental behavioral manipulations, including handling, courtship, and aggression, affect the number of brain mast cells. These manipulations often elicit behavioral arousal induced through psychological stressors, and factors affecting mast cell numbers in the brain are likely to be neurophysiologically important. The responses of brain mast cells to a number of local stimuli may regulate neuroimmune interactions, possibly contributing to the integration of behavior with neural activity.

Mast cells contain multiple chemicals which possibly affect sleep/wake regulations, such as histamine, prostaglandin D_2_ (PGD_2_), and tumor necrosis factor alpha (TNFα)  [5-7]. Histamine is one of the most potent neurotransmitters affecting the modulation of animal behavior. Brain histamine localizes in both mast cells and histamine neurons, with the mast cells storing approximately 50% of it’s whole brain levels, since brain histamine levels in mast cell deficient mice are approximately 50% of that in wild-type mice [17]. Neuronal histamine is released in the brain from histamine neurons located in the tuberomammillary nucleus (TMN) in the posterior hypothalamus, and the histaminergic neurons project to almost all regions of the mammalian brain [18-20]. Histaminergic neurons discharge selectively during wakefulness, and that arousal is provoked by the enhancement of histaminergic transmission with many excitatory inputs, including hypocretin/orexin which directly depolarizes histaminergic neurons of TMN [21]. On the contrary, slow-wave sleep (SWS) is promoted by the inhibition of H1 receptor antagonist in cats and rodents [22-25]. In addition, mice lacking histamine due to disruption of the histidine decarboxylase (HDC), a key enzyme for histamine biotsynthesis, show deficit in wakefulness and interest in new environments [26]. Although mast cell-derived histamine may also be involved in sleep/wake regulation, this has never been studied. 

The availability of *Kit* ^*W/W-v*^ mouse mutants provided a powerful genetic tool for the in vivo analysis of the role of mast cells, because these animals lack detectable mast cells in their bodies [27]. In this study, we examined the role of brain mast cells, focusing especially on the histaminergic system, using mast cell deficient mice and neurophysiological and neuorpharmacological manipulations. We explored the possibility that mast cells in the brain contribute to the modulation of sleep/wake and arousal behavior, including food-seeking behavior and mood states.

## Materials and Methods

### Animals

Eight-week old male mast cell deficient mice (WBB6F_1_/J-*Kit^W^*/*Kit* ^*W-v*^; W/W^v^, [28]) and their wild-type littermates (WT) were obtained from the Jackson Laboratory (Bar Harbor, ME). W/W^v^ mice do not contain detectable mast cell populations anywhere in the entire body, including the brain [27]. Food and water were available *ad libitum*. A 24-hour light-dark cycle (lights on for 12 hours, off for 12 hours) was maintained throughout the study (lights on at zeitgeber time [ZT] = 0 at 07:00 am). Room temperature was maintained at 24 ± 1°C throughout experimentation. The entire study was approved by, and conducted in accordance with, The Stanford University Administrative Panel on Laboratory Animal Care guidelines.

### Sleep recordings and analysis

Electroencephalogram (EEG)/electromyogram (EMG) implantation surgery was performed as described before [29]. The digitally filtered (30 Hz Low Pass Filter for EEG; 10–100 Hz Band Pass Filter for EMG) EEG and EMG signals were captured at 128 Hz using a sleep recording system (Vital Recorder, Kissei Comtec, Matsumoto, Japan). Off-line sleep scoring was done on the computer by visual assessment of the EEG and EMG activities using the SleepSign analysis program (Kissei Comtec, Matsumoto, Japan). The vigilance states were classified for each 10-second epoch as wakefulness, rapid-eye movement sleep (REM), or SWS. The EEG power spectrum in the epoch that was scored as SWS was calculated by Fast Fourier Transform using the SleepSign analysis program. The EEG delta frequency band was set at 0.5 - 4.0 Hz. The delta power was normalized and described as a percentage of the total power (0.5 - 30 Hz), and the data was averaged at hourly intervals.

In order to evaluate change in locomotor activity and core body temperature, an implant telemetry device (G2 E-Mitter, Mini Mitter, Bend, OR) was implanted in the abdominal cavity of each mouse [29].

### Pharmacological treatments and injection procedure

Drug injections during the dark period were done under a dim, red light. Cannulae were implanted intracerebroventricularly (icv) at the time of the surgery for EEG and EMG, as described before [30]. Either compound 48/80 (C48/80; 1, 5, 10 μg dissolved in 1.0 μl saline, Sigma Chemical Co., St. Louis, MO, USA), a selective histamine releaser from mast cells, or the vehicle (saline) was injected icv and slowly over the course of 1 minute, using a Hamilton microsyringe at ZT14. In order to evaluate the pharmacological effects of histamine H1 receptor antagonists, triprolidine (5, 10, and 20 mg/kg), mepyramine (0.25, 1, and 4 mg/kg), and diphenhydramine (2.5, and 10 mg/kg) were intraperitoneally (ip) administered to each animal at ZT14. Thioperamide (1.25, 5, and 10 mg/kg, ip), a H3 receptor antagonist, and alpha-FMH (50 and 100 mg/kg, ip), a histidine decarboxylase inhibitor, were also administered to each animal at ZT2 and ZT12 respectively. Sleep data for the 6 hours following administrations were captured and analyzed.

### Procedure for sleep deprivation (SD) and food deprivation

To evaluate sleep homeostasis, mice were sleep-deprived for 6 hours, between ZT0 and ZT6, using a small, soft brush to touch the back of the mouse several times when it appeared to be sleepy. At ZT6, SD was terminated, and the EEG and EMG were recorded for an 18-hour period of uninterrupted recovery sleep. For food deprivation, food pellets were carefully removed from the recording cages at the onset of the dark period (ZT12), and the EEG and EMG were recorded for 24 hours.

### In vivo microdialysis

WT and W/W^v^ mice were implanted with stainless steel guide cannulae for microdialysis and drug injection under general anesthesia with 3% isoflurane. The microdialysis cannula was implanted into the left lateral ventricle (AP -0.5 mm; ML 1.2 mm; V 1.5 mm to bregma) or thalamus (AP -0.5 mm; ML 3.2 mm, Angle 30°; V 4.0 mm to bregma). The cannula for drug injection was implanted obliquely into the right ventricle (AP -2.2 mm; ML 0.9 mm; V 2.5 mm, Angle 30°, to bregma). After 2 weeks of recovery, a microdialysis probe with a 2 mm-long semipermeable membrane (Eicom, Kyoto, Japan) was inserted into the lateral ventricle in conscious mice, 3-4 hours prior to the microdialysis experiment. The microdialysis lines were connected to the probe and pump, and were continuously perfused at a rate of 1 μl/min with a Ringer solution. Dialysate was collected every 30 minutes from 2 hours before to 6 hours after drug injection. After sampling the baseline for 2 hours, C48/80 (5 μg, icv) was injected. 

Histamine content in the CSF was determined by the HPLC-fluorometry technique established by Yamatodani et al. [31]. Dialysate samples (30 μl) were injected directly into a column packed with the TSKgel SP2SW Cation Exchanger (150 mm × 3.0 mm i.d., Tosoh, Tokyo, Japan). The histamine was eluted with 0.25 M potassium phosphate at a flow rate of 0.3 ml/min, was post-labeled with o-phthalaldehyde in an alkaline condition, and was then detected fluorometrically in an F1080 Fluorometer (Hitachi, Tokyo, Japan).

### Anxiety and depression levels

To evaluate anxiety and depression levels, elevated plus maze and forced swim tests were conducted in WT and W/W^v^ mice during the light phase. For behavioral tests, we used another group of mice, which did not undergo surgery for sleep recording. The elevated plus maze test was performed as described before [29]. Increased time spent in closed arms was an indication of increased anxiety. 

In the forced swim test, the mice were subjected to swim sessions in individual glass cylinders (height 40 cm, diameter 20 cm) containing water that was 15 cm deep and at 25°C. Mice were tested for 6 minutes, and immobility, defined as no activity for at least 2 seconds, was measured during the last 4 minutes of the test. Increased duration of immobility was an indication of depression-like behavior. The effects of imipramine (10 and 20 mg/kg, ip) and saline on depression-like behavior were evaluated in WT and W/W^v^ mice.

### Statistics

Results are expressed as the mean ± SEM. The data was analyzed by repeated measurement of two-way ANOVA, followed by a student’s t-test or paired t-tests for sleep recording at baseline, SD, food deprivation, microdialysis results, locomotor activity and elevated plus maze tests. One-way ANOVA followed by Scheffe’s post-hoc test was applied for dose-dependent actions of C48/80, H1 blockers, and imipramine. For all comparisons, the criterion for significance was p < 0.05 (2-tailed).

## Results

### Baseline and SD

**Figure 1:**
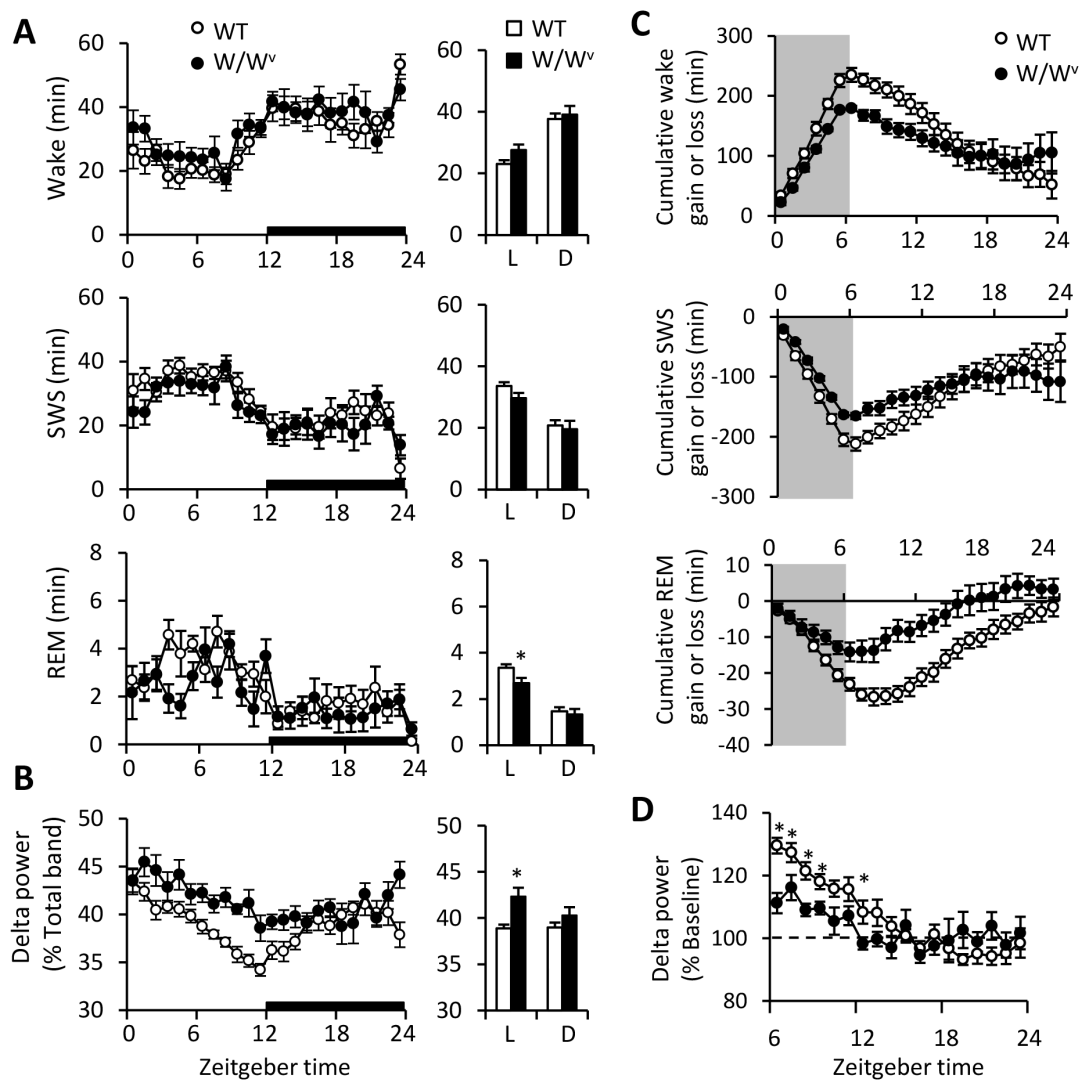
Baseline sleep/wake characterizations of WT and W/W^v^ mice. (A) Time course for amount of wake (top) and SWS (middle) was not different between the two genotypes. Amount of REM (bottom) was slightly decreased in W/W^v^ mice during the light phase (L; ZT0-12) but was not during the dark phase (D; ZT12-24). (B) EEG delta power in SWS was significantly higher in W/W^v^ mice during the light phase (L; ZT0-12). The bar graphs indicate the averaged data for each 12-hour period across ZT0-12 (L) and ZT12-24 (D). (C) Cumulative sleep/wake loss and gain compared with baseline conditions for the sleep deprivation experiment. Sleep deprivation began at ZT0 and ended at ZT6 (the shadow areas). (D) The rebound response of EEG delta power in SWS after sleep deprivation was attenuated in W/W^v^ mice. Data is expressed as the percent change from the baseline value (dotted line) at the same time. Amount of sleep/wake and EEG delta power was averaged at hourly intervals. *p < 0.05, **p < 0.01, WT versus W/W^v^. All data is expressed as mean ± SEM (n = 8/group).

Body weight and food intake were identical in WT and W/W^v^ mice (data not shown). Separate histological examinations of the brains of WT and W/W^v^ mice revealed that mast cells were observed in the brains of WT mice, especially in periventricular organs around the hippocampus, while mast cells were not seen in the brains of W/W^v^ mice (Figure S1). Sleep/wake patterns of W/W^v^ mice during wake and sleep were similar to those of WT mice (Figure 1A), and no abnormal EEG activities such as proximal spikes and waves were seen in W/W^v^ mice. W/W^v^ mice showed increased EEG delta power in SWS in the light phase (*t* = 2.61, *P*<0.05, Figure 1B), suggesting that W/W^v^ mice may have an altered function of sleep homeostasis. To evaluate this possibility, mice were deprived of sleep for 6 hours. During the recovery period after SD, WT mice showed an increased amount of SWS and REM, while this rebound response was much smaller in W/W^v^ mice (Figure S2). Cumulative sleep/wake gain and loss data shows that W/W^v^ mice gain less in SWS during the recovery period (*F*_1,23_ = 36.61, *P*<0.0001, Figure 1C) than WT mice. In addition, W/W^v^ mice showed an attenuated rebound response in EEG delta power in SWS (*F*_1,17_ = 11.10, *P*<0.0001, Figure 1D). These results suggest that there is no significant change in sleep structure of W/W^v^ mice, but their sleep homeostasis is altered.

### Pharmacological tests

**Figure 2:**
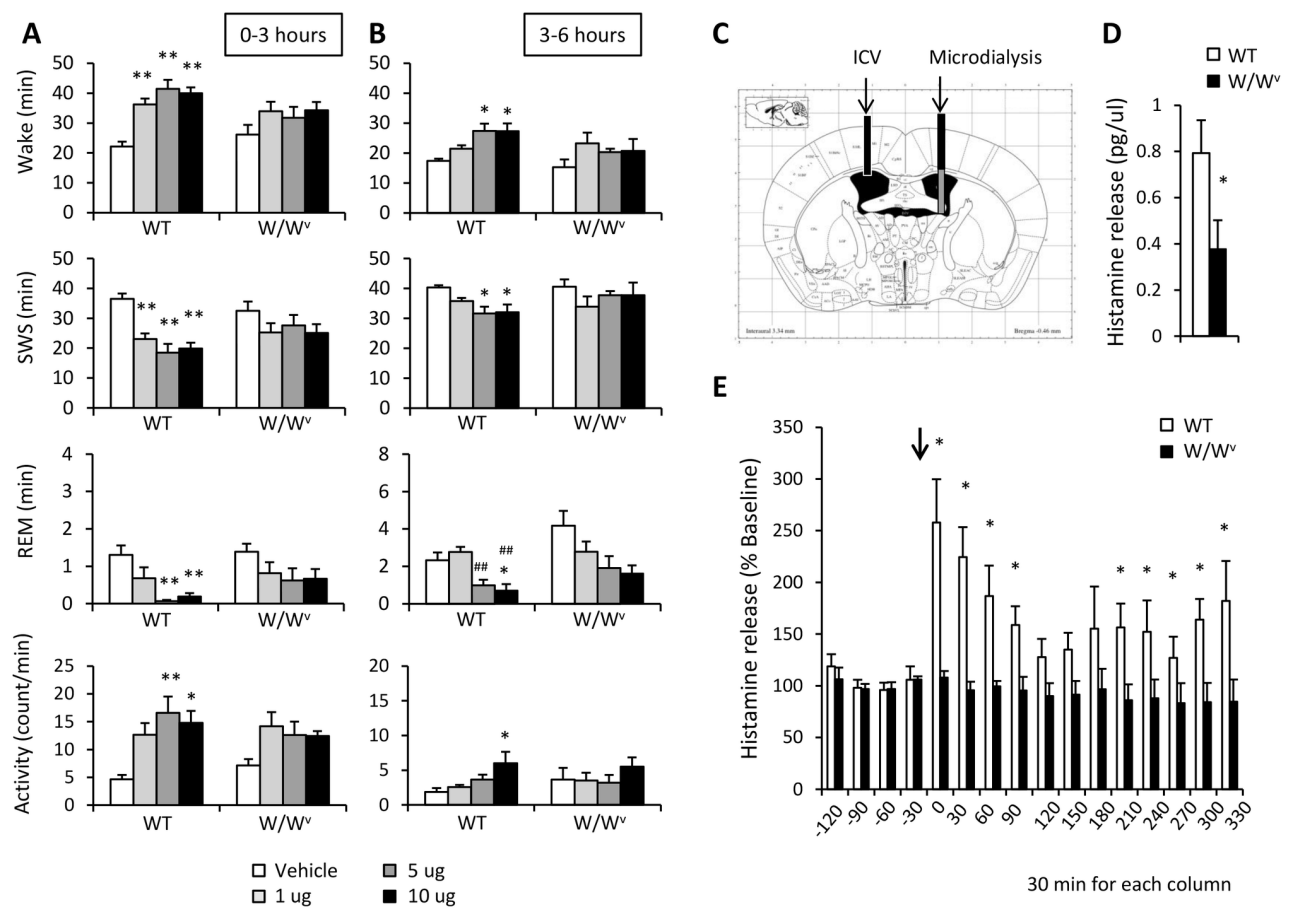
The effects of icv injection of compound 48/80 (C48/80) on sleep/wake and locomotor activity in WT and W/W^v^ mice. Icv injection of C48/80 promoted wakefulness for both (A) 0-3 hours and (B) 3-6 hours after the injection in WT, but not in W/W^v^ mice. Amount of sleep/wake and locomotor activity was averaged at hourly intervals. *p < 0.05, **p < 0.01, versus vehicle; ^＃＃^p < 0.01, versus 1 μg. Data is expressed as mean ± SEM (n = 8/group). (C) A schematic representation of the lateral ventricle sections adopted from Franklin and Paxinos [59]. Black bars indicate the placement of the guide cannulae for icv injection of C48/80 (5 μg) and microdialysis probe. Microdialysis membranes, indicated as gray bars, were inserted into lateral ventricle. (D) Baseline histamine levels in the lateral ventricle of WT mice were higher than that of W/W^v^ mice (WT: 0.792 ± 0.174 pg/μl, W/W^v^: 0.378 ± 0.056 pg/μl). (E) Histamine levels in the lateral ventricle increased by C48/80 stimulation in WT mice, while it did not change in W/W^v^ mice. Data is expressed as the percent change from the baseline value (average value for 90 minutes before the injection) of each group. *p < 0.05, versus WT mice. Histamine levels are expressed as mean ± SEM (n=6-7/group). Each column represents the histamine level for 30 minutes. The arrow (↓) indicates the time of the C48/80 injection.

Next, we examined the contribution of histamine from brain mast cells to sleep/wake regulations, by administering C48/80, a histamine releaser from mast cells. Icv injection of C48/80 increased the amount of wakefulness and locomotor activity in WT mice (mice injected with 5 μg C48/80 showed 187 % wakefulness (*F*_3,28_ = 15.83, *P*<0.0001) and 358 % activity (*F*_3,28_ = 6.16, *P*<0.01) of vehicle-injected control), while it had no effect on that of W/W^v^ mice (Figure 2A). This effect of C48/80 on sleep/wake and activity continued for over 6 hours after the injection (mice injected with 10 μg C48/80 showed 158 % (wakefulness; *F*_3,27_ = 5.98, *P*<0.01) and 196 % (activity; *F*_3,28_ = 3.67, *P*<0.05) of vehicle-injected control, Figure 2B). Basal histamine levels in the brain (in and adjacent to the lateral ventricle) of W/W^v^ mice was decreased to 48 % of that of WT mice at baseline (*t* = -2.273, P<0.05, Figure 2D). These results are consistent with previous histamine measurements in mast cell deficient mice [17] and our histology results (Figure S1). The histamine release in the lateral ventricle region increased immediately after the injection of C48/80 in WT mice, but did not increase in W/W^v^ mice (Figure 2E). 

**Figure 3:**
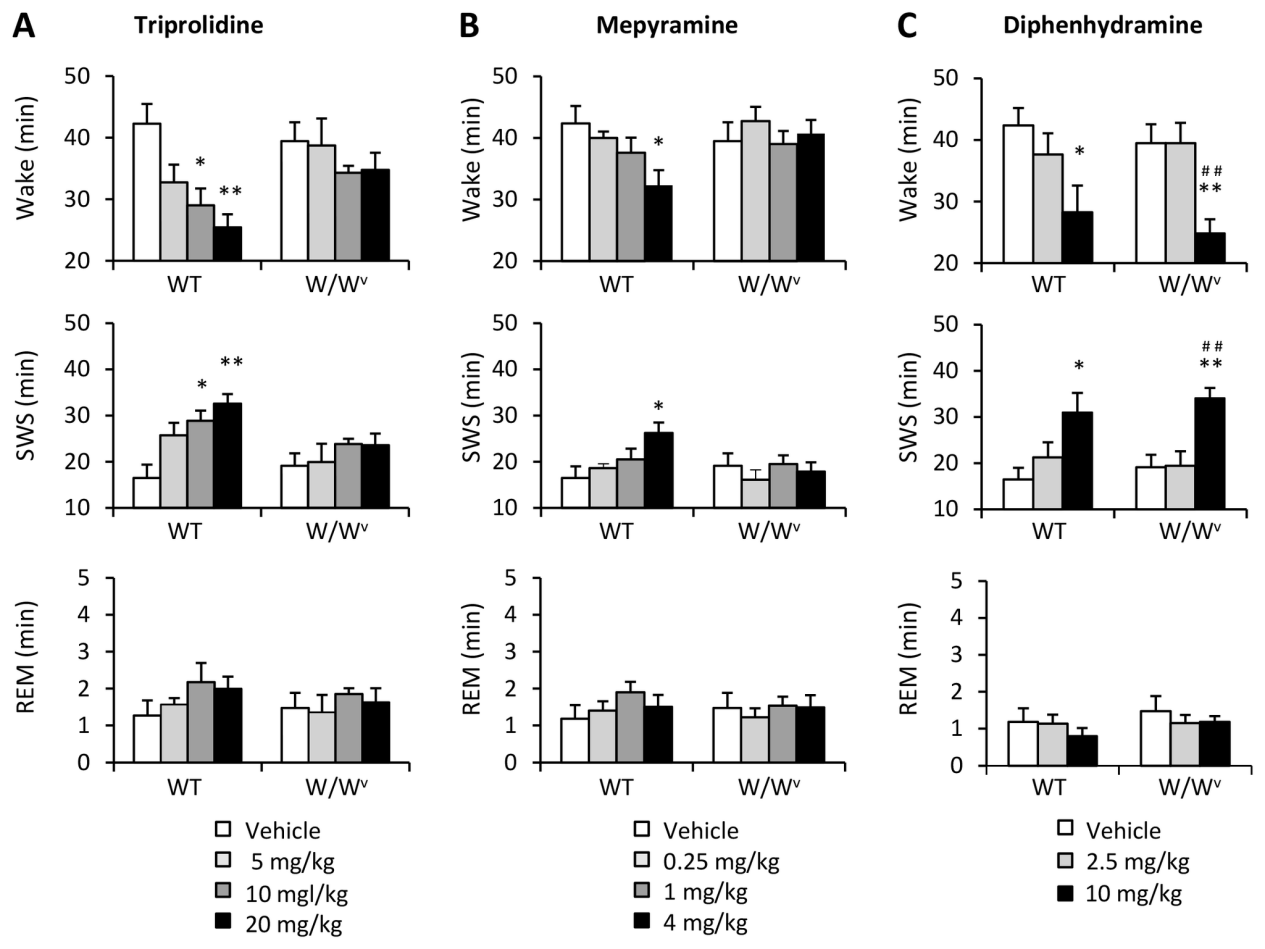
The effects of histamine H1 receptor antagonists on the amount of sleep/wake in WT and W/W^v^ mice (A) Triprolidine and (B) mepyramine increased SWS in only WT mice, while (C) diphenhydramine affected SWS in both WT and W/W^v^ mice. Amount of sleep/wake was averaged at hourly intervals for 3 hours after drug injection. *p < 0.05, **p < 0.01, versus vehicle; ^#^p < 0.05, ^##^p < 0.01, versus low (2.5 mg/kg diphenhydramine) dose. All data is expressed as mean ± SEM (n = 7-8/group).

**Figure 4:**
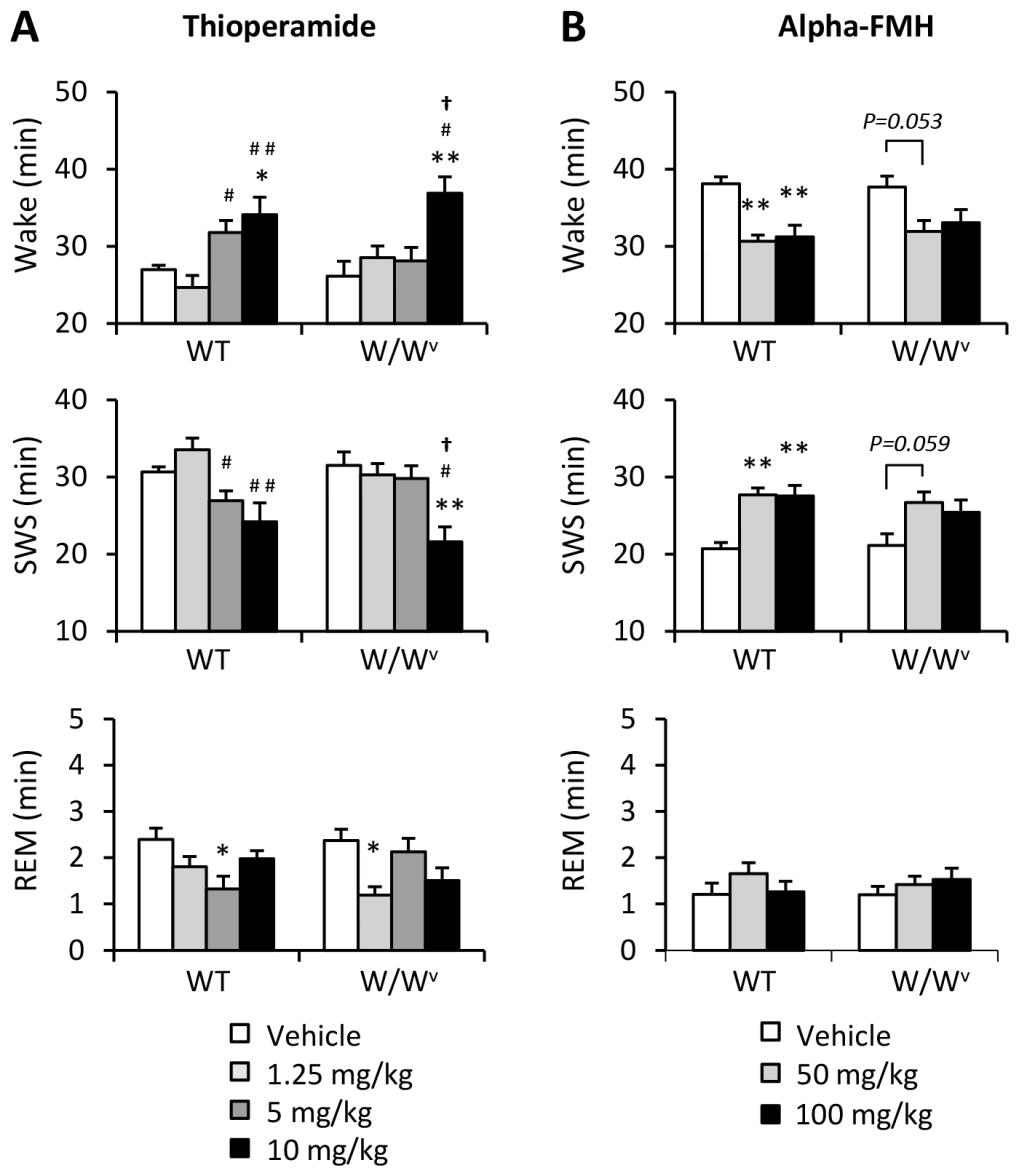
The effects of thioperamide and alpha-FMH on the amount of sleep/wake in WT and W/W^v^ mice. (A) Injection of thioperamide (ip), a H3 antagonist, at a high dose (10 mg/kg) increased wakefulness in both WT and W/W^v^ mice for 3 hours. (B) In contrast, injection of alpha-FMH (ip), a HDC blocker, at medium (50 mg/kg) and high (100 mg/kg) doses decreased the amount of wakefulness in WT mice. Amount of sleep/wake was averaged at hourly intervals. *p < 0.05, **p < 0.01, versus vehicle; ^#^p < 0.05, ^##^p < 0.01, versus low (1.25 mg/kg thioperamide) dose; **^†^**p < 0.05, versus middle (5mg/kg thioperamide) dose. All data is expressed as mean ± SEM (n = 6-8/group).

To investigate whether histamine from brain mast cells that are active in the basal state is involved in sleep/wake regulation, we tested the effects of sleep-inducing H1 blockers (triprolidine, mepyramine, and diphenhydramine, ip) on sleep/wake. In WT mice, injection of 20 mg/kg triprolidine and 4 mg/kg mepyramine decreased the amount of wakefulness (60 % (triprolidine; *F*_3,24_ = 6.84, *P*<0.01) and 76 % (mepyramine; *F*_3,25_ = 3.41, *P*<0.05) of vehicle-injected control), and increased SWS for 3 hours after the injection (198 % (triprolidine; *F*_3,24_ = 7.51, *P*<0.001) and 159 % (mepyramine; *F*_3,25_ = 3.83, *P*<0.05) of vehicle-injected control), while the sleep-inducing effects of these H1 blockers were not observed in W/W^v^ mice (Figure 3A and 3B). A high dose (10 mg/kg) of diphenhydramine, another type of H1 blocker, increased SWS in WT (188 % of vehicle-injected control; *F*_2,21_ = 4.72, *P*<0.05, Figure 3C). A high dose (10 mg/kg) of diphenhydramine also increased sleep in W/W^v^ mice (178 % of vehicle-injected control; *F*_2,21_ = 9.80, *P*<0.001, Figure 3C). These results suggest that sleep-inducing effects of H1 blockers may be partially mediated by blockades of histamine released from mast cells. On the other hand, wake-promoting effect of thioperamide (10 mg/kg), a H3 autoreceptor antagonist, was shown in both WT (126 % of vehicle-injected control; *F*_3,26_ = 7.41, *P*<0.001) and W/W^v^ (141 % of vehicle-injected control; *F*_3,23_ = 6.44, *P*<0.01) mice (Figure 4A). Injection of 50 mg/kg of alpha–FMH, a histamine synthesis inhibitor, decreased wakefulness in WT (81 % of vehicle-injected control; *F*_2,18_ = 14.36, *P*<0.0001), and this tendency was also shown in W/W^v^ mice (85 % of vehicle-injected control, Figure 4B). 

### Food seeking behavior

**Figure 5:**
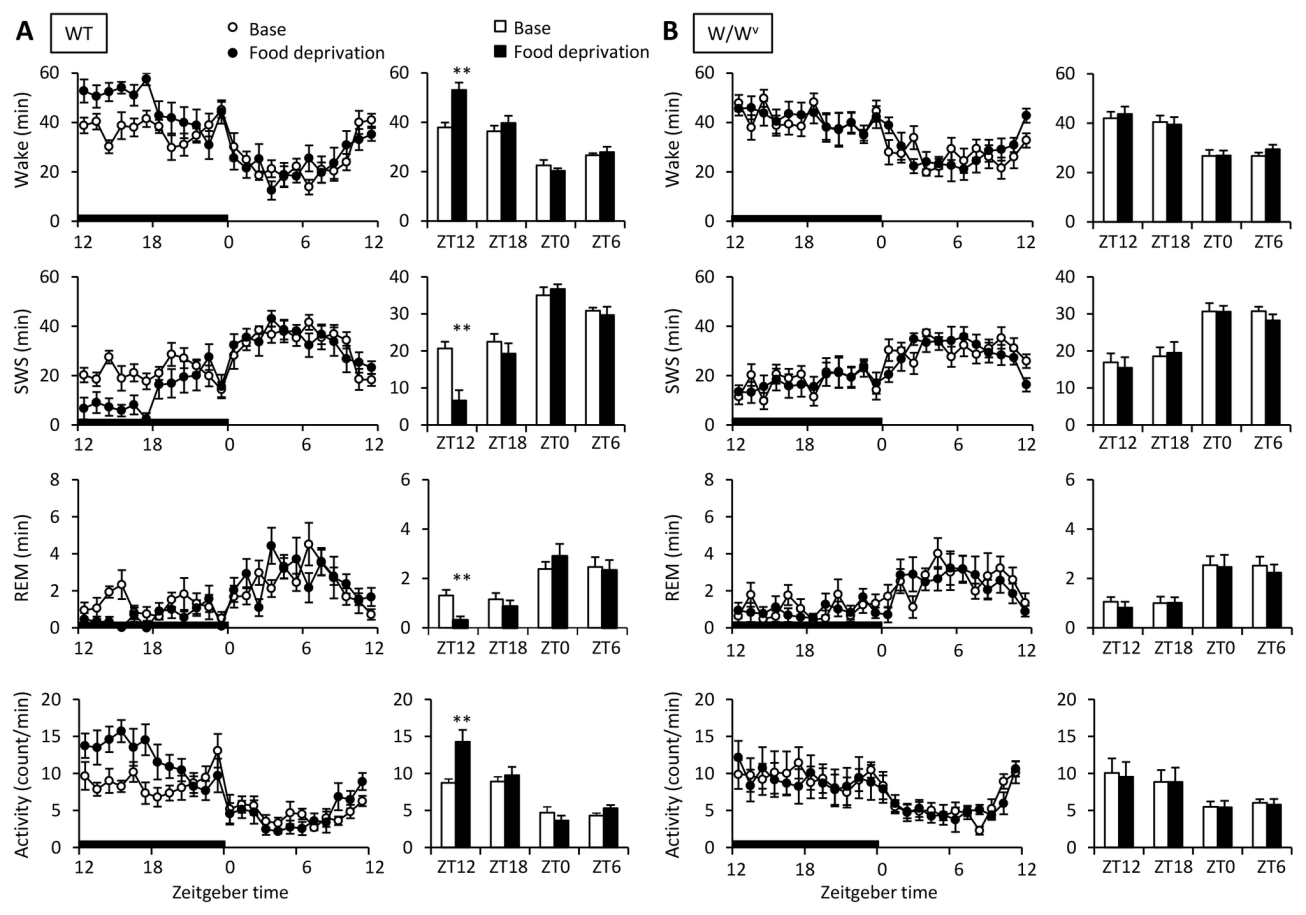
The effects of food deprivation on the amount of sleep/wake in WT and W/W^v^ mice. During food deprivation for 24 hours, (A) WT mice showed food seeking behavior in the early half of the dark phase. (B) However, food seeking behavior was not observed in W/W^v^ mice. Food deprivation began at ZT12 and ended at ZT12 the next day (closed circles and columns). In the bar graphs, data was averaged at each 6-hour interval at ZT12-18 (ZT12), ZT18-24 (ZT18), ZT0-6 (ZT0), and ZT6-12(ZT6). Amount of sleep/wake and locomotor activity was averaged at hourly intervals. *p < 0.05, **p < 0.01, versus baseline. All data is expressed as mean ± SEM (n = 7-8/group).

It is well established that food deprivation enhances wakefulness in rodents due to the arousal response through increased food-seeking behavior [32-35]. Also, histamine neurotransmission is elevated during food deprivation [36,37]. We investigated whether mast cells, or histamine from mast cells, are involved in the arousal response during food deprivation. When WT mice were deprived of food, they showed a marked increase in wakefulness (*t* = -7.95, *P*<0.001) and locomotor activity (*t* = -4.13, *P*<0.01) during the first half of the dark period (Figure 5A). Interestingly, the increase in wakefulness and locomotor activity was completely abolished in W/W^v^ mice (Figure 5B). These results suggest that mast cells may regulate arousal when mice are faced with a negative energy balance due to reduced food availability.

### Anxiety and Depression Levels

**Figure 6:**
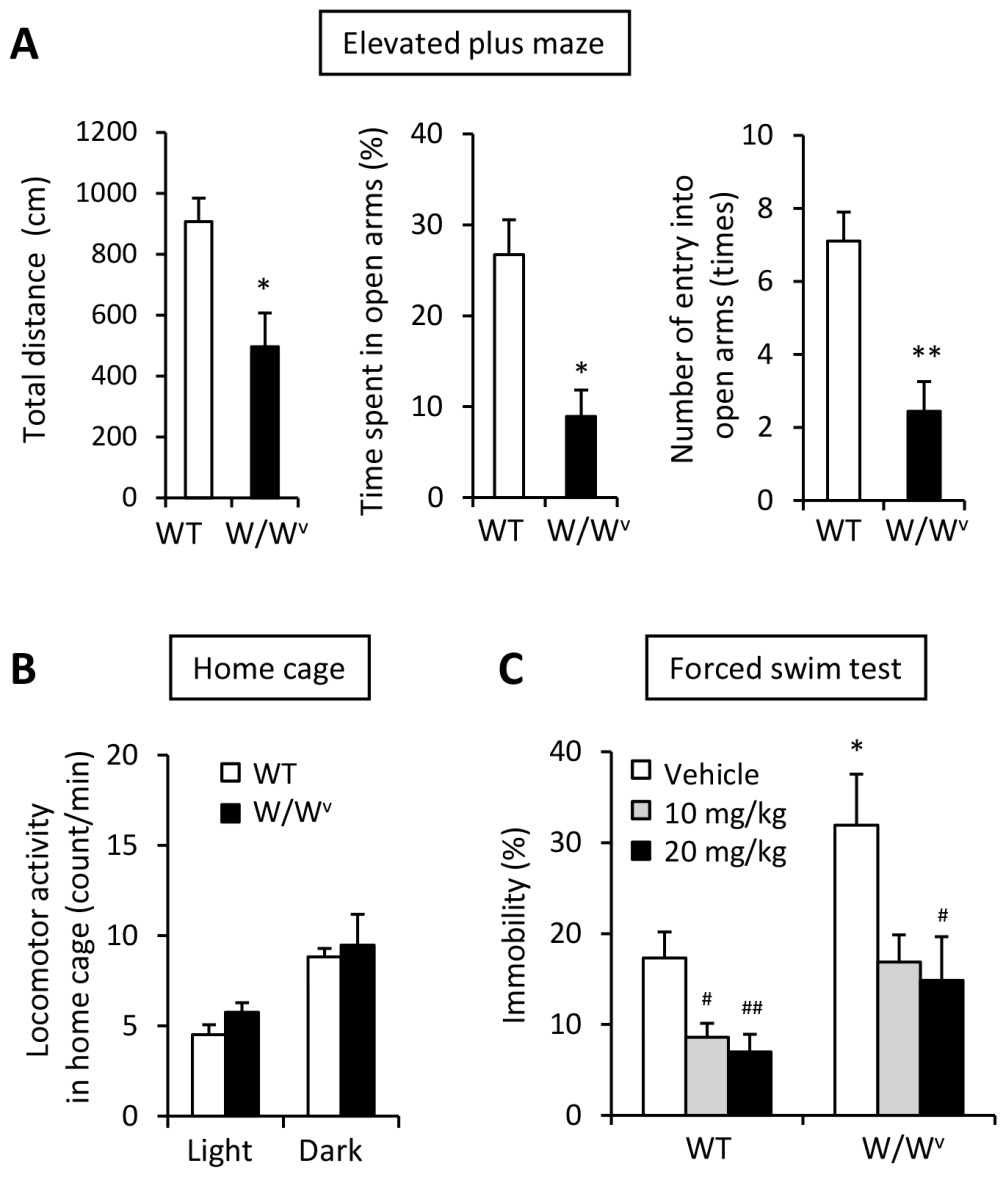
Increased anxiety- and depression-like behavior in W/W^v^ mice. (A) Anxiety-like behavior in an elevated plus maze in WT and W/W^v^ mice at baseline. Total distance (left panel), time spent in open arms (center panel) and number of instances of entry into open arms (right panel) was decreased in W/W^v^ mice compared to that of WT mice. (B) Locomotor activities in home cage were not different between the two groups. (C) Depression-like behavior increased significantly in W/W^v^ mice. The increased depression-like behavior was ameliorated by imipramine treatment (ip). Imipramine was administrated into WT and W/W^v^ mice 30 minutes before a forced swimming test. *p < 0.05, **p < 0.01, versus WT. ^#^p < 0.05, ^##^p < 0.01, versus vehicle. All data is expressed as mean ± SEM (n = 6-8/group).

Next, we evaluated changes in anxiety and depression levels in W/W^v^ mice to confirm the effect of mast cells on mood state. In W/Wv mice, total distance travelled in the elevated plus maze was decreased compared to that of WT mice (*t* = -3.13, *P*<0.05, Figure 6A), although there was no difference in locomotor activity in the home cages between the genotypes (Figure 6B). In the elevated plus maze, W/W^v^ mice had fewer entries (*t* = -3.72, *P*<0.05) and spent less time in open arms (*t* = -4.10, *P*<0.001) compared to WT mice (Figure 6A). In addition, W/W^v^ mice showed greater immobility during the forced swim test (Figure 6C). These enhanced depression-like behaviors observed in W/W^v^ mice were recovered through the injection of imipramine (*F*_2,24_ = 4.12, *P*<0.05), a tricyclic antidepressant with serotonin and noradrenaline uptake inhibitions (Figure 6C), suggesting that mast cells contribute to the regulation of mood state.

## Discussion

This is the first study demonstrating how mast cells in the brain play a role in the regulation of sleep and other behaviors. W/W^v^ mice have altered sleep and behavioral responses to sleep and food deprivation and pharmacological manipulation, compared to WT mice, although W/W^v^ mice show the same sleep/wake architecture under baseline conditions. 

In the present study, we found that mast cell deficient mice have an enhanced EEG delta power in SWS during the light phase and a smaller rebound response to 6-hour sleep deprivation. The systemic administration of a H1 blocker enhances EEG delta power in SWS of mice [38], and H1 receptor knockout mice show increased EEG frequency range of upper delta through lower theta in SWS [22]. Furthermore, we have previously observed a marked increase in CSF histamine after 6-hour sleep deprivation in rats [39], suggesting that central histamine is involved in the regulation of sleep homeostasis and increased histamine may counterbalance sleep propensity.

Because C48/80 is a selective histamine releaser from mast cells, administration into the lateral ventricle is expected to evoke physiological responses regulated by histamine released from mast cells. Indeed, we showed that icv administration of C48/80 produces a significant increase in wakefulness and locomotor activity in WT mice, but the effects were completely abolished in W/W^v^. This data was consistent with the microdialysis results, which showed that histamine release in the lateral ventricle increased immediately after the injection of C48/80 in WT mice but not in W/W^v^ mice. These results demonstrated that histamine released from brain mast cells by pharmacological manipulation is wake-promoting. When the microdialysis probe was inserted into the thalamus, histamine increase was not observed in either type of mouse (Figure S3). Icv injected drugs may not reach the thalamus. Alternatively, mast cell-derived histamine may be involved primarily in volume transmission known in other sleep regulatory neurotransmitters such as PGD_2_ [40,41], especially since brain mast cells are enriched in leptomeninges and circumventricular organs, which are crucial structures for volume transmission [7]. 

Histamine H1 receptors mediate excitatory actions on whole brain activity, and H1 receptor antagonists increase SWS [21-23,25]. We observed that the effect of sleep-inducing H1 blockers (triprolidine and mepyramine) was attenuated in W/W^v^ mice, although a high dose of diphenhydramine also induced sleep in W/W^v^ mice. The histamine H1 receptor is a seven transmembrane-spanning receptor coupled with G protein Gq/11 and its activation leads to phospholipase C stimulation. Diphenhydramine has lower affinity for H1 receptors and higher affinity for muscarinic cholinergic receptors compared with triprolidine and mepyramine [42,43]. Diphenhydramine also acts as an intracellular sodium channel blocker [44], a reuptake blocker of serotonin [45], and potentiator of analgesia induced by morphine in rats [46], and all of these non-histaminergic effects likely modify the sleep-inducing effects of diphenhydramine. Our microdialysis data suggests that non-neuronal histamine from mast cells is likely to be wake-promoting. Since sleep-inducing effects seen in two out of three H1 antagonist tested are significantly attenuated in W/Wv mice, and since diphenhydramine possess large varieties of pharmacological actions, we interpreted that the sleep inducing effects of H1 antagonists may be partially mediated by blockades of histamine release from mast cells. Conversely, sleep inducing effects of the histamine synthesis inhibitor (alpha–FMH) and wake-promoting effects of an H3 autoreceptor antagonist (thioperamide) are retained in W/W^v^ mice. However, this result is still consistent with our interpretation that brain mast cells regulate sleep, since large amounts of histamine are pre-packed in the mast cells and it is unlikely that alpha–FMH (i.e. a histamine synthesis inhibitor) acutely reduces histamine release from mast cells [47]. Similarly, H3 antagonists act on terminals of histamine neurons, and it is unlikely that increased histamine release from histaminergic neuronal terminals [48] (i.e., presynaptic autoreceptors) affects histamine release from mast cells.

The most profound phenotype of the mast cell deficient mice is the lack of increase in wakefulness/locomotor activity during food deprivation. Several recent reports have demonstrated that hypothalamic neuronal histamine, and possibly brain-derived mast cell histamine, are involved in the regulation of food intake. Rats fasting for 24 hours showed increased hypothalamic histamine content [37], and neuronal glucoprivation enhanced hypothalamic histamine turnover [36]. In addition, the hypothalamic histaminergic system is activated during feeding-related motivated behavior, which activates arousal systems [49,50]. Concerning the histamine released from brain mast cells, it was also reported that icv injection of C48/80 suppressed food intake of neonatal chicks [51]. In the present study, W/W^v^ mice showed a lack of food-seeking behavior during food deprivation, although we did not measure histamine levels during food deprivation. These results suggest that histamine released from brain mast cells may modulate homeostatic control of energy balance in response to energy deficiency. Since W/W^v^ mice showed normal food intake at baseline, mast cells or substances released from mast cells would be necessary to maintain enhanced arousal to react in the situation of energy deficiency.

Finally, we found that mast cells can contribute to modulation of anxiety and depression-like behavior, the two most common psychiatric symptoms seen in humans. Histamine has been reported to have both anxiolytic and anxiogenic effects [19], because H1 antagonists have an anxiogenic effect while H2 receptor antagonists have an anxiolytic effect in mice [52]. Our results were consistent with the previous study in which mast cell deficient mice (*Kit* ^*W-sh/W-sh*^, another type of *Kit* mutant mice lacking mast cells) showed increased anxiety [53]. In the present study, we further observed that W/W^v^ mice exhibited depression-like behavior and the symptoms were recovered through injection of imipramine, an inhibitor of serotonin and noradrenaline uptake. Serotonin and selective serotonin reuptake inhibitors (SSRI), which increase serotonin signaling, have been known to decrease anxiety and depression [54]. Therefore, mast cell-derived histamine may also modulate mood state, along with interactions with other mast cell derived mediators including serotonin. 

Although most of the results presented are novel and may have significant impacts on clinical and basic neuroscience, the limitations of the study should be motioned. In contrary to the detailed descriptions of excitatory (hypocretin/orexin, glutamine, acetylcholine, and serotonin) and inhibitory (gamma-aminobutyric acid and galanin) inputs to neuronal histaminergic neurons, the mechanism triggering histamine release from mast cells in the regulation of sleep/wakefulness and feeding behavior is not known. Available findings suggest that diffusion through the brain extracellular fluid of neurotransmitters released, including CRH, catecholamines, neuropeptide Y  [8,9,14-16] may activate extrasynaptic receptors on the mast cells, but these need to be investigated. 

Mast cell-deficient *Kit* ^*w/w-v*^ mice have been commonly used as an animal model for the analysis of mast cell function [27]. However, *c-kit* mutations (*Kit* ^*W/W-v*^ and *Kit* ^*W-sh/W-sh*^), alter the *c-kit* coding region, and thereby cause varying degrees of impairment to intrinsic *c-kit* receptor function. For example, *Kit* ^*W/W-v*^ mice develop dermatitis, gastric ulcers, anemia, and sterility [55-57]. Since a new mast cell deficient mice model, with a conditional knockout of the *Mcl-1* (myeloid cell leukemia sequence 1) in the carboxypeptidase A3 (*Cpa3*) expressing cells, has recently been introduced [58], it is critical to replicate our results using these non-kit mutant mast cell deficient mice,

In summary, the results of our study provide evidence of the neurobehavioral importance of brain mast cells. Our data suggests that non-neuronal histamine from brain mast cells is involved in the sleep/wake regulation, and more specifically, contributes to the regulation of food-seeking behavior, the most fundamental mammalian behavior since time immemorial. Our results also open new avenues for the role of brain mast cells in neuropsychiatric research, as many other mast cell derived mediators likely regulate neurobehaviors in health and diseases.

## Supporting Information

Figure S1 **Connective tissue type mast cells in the brain of WT and W/W^v^ mice.** Toluidine blue-stained mast cells using toluidine blue solution (0.05 %, pH2.5, Wako, Japan) are shown in the right side (A-D) and the left side (E-H) of the brain in WT (A, B, E and F) and W/W^v^ (C, D, G and H) mice. B, D, F and H show enlargements from the boxed regions at left (A, C, E and G) respectively. Scale bars indicate 100 μm. Abbreviations: dentate gyrus (DG), dorsal 3rd ventricle (D3V), thalamus (Thal), field CA3 hippocampus (CA3).
(TIF)

Figure S2 **Rebound response after 6-hour sleep deprivation in (**A**) WT and (**B**) W/W^v^ mice.** Time courses of sleep/wake for every hour during and after sleep deprivation are shown in the left panel. Sleep deprivation began at ZT0 and ended at ZT6 (the shadow areas). In the bar graphs (right panel), average amount of sleep/wake was calculated for each of the 6-hour periods across ZT6-12 (ZT6), ZT12-18 (ZT12), and ZT18-24 (ZT18). *p < 0.05, **p < 0.01, WT versus W/W^v^ mice. All data is expressed as mean ± SEM (n = 8/group).
(TIF)

Figure S3 **The effects of icv injection of compound 48/80 (C48/80) on histamine levels in WT mice.** (A) A schematic representation of thalamus and lateral ventricle sections adopted from Franklin and Paxinos (Franklin and Paxinos, 2008). Black bars indicate the placement of the guide cannulae for icv injection of C48/80 and microdialysis probe. Microdialysis membranes, indicated as gray bars, were inserted into the thalamus (top panel) and lateral ventricle (bottom panel). (B) Extracellular histamine levels in the thalamus (top panel) and lateral ventricle (bottom panel) were measured by mast-cell stimulation of 5 μg C48/80 in each WT mouse (n=3). Each column represents the histamine levels for 30 minutes. The arrow (↓) indicates the time of the C48/80 injection.
(TIF)
