# Supplementary figures and images for: Histamine from Brain Resident MAST Cells Promotes Wakefulness and Modulates Behavioral States
Source: PLoS One. 2013 Oct 18;8(10):e78434. doi: 10.1371/journal.pone.0078434 (PMC3800008; doi:10.1371/journal.pone.0078434)

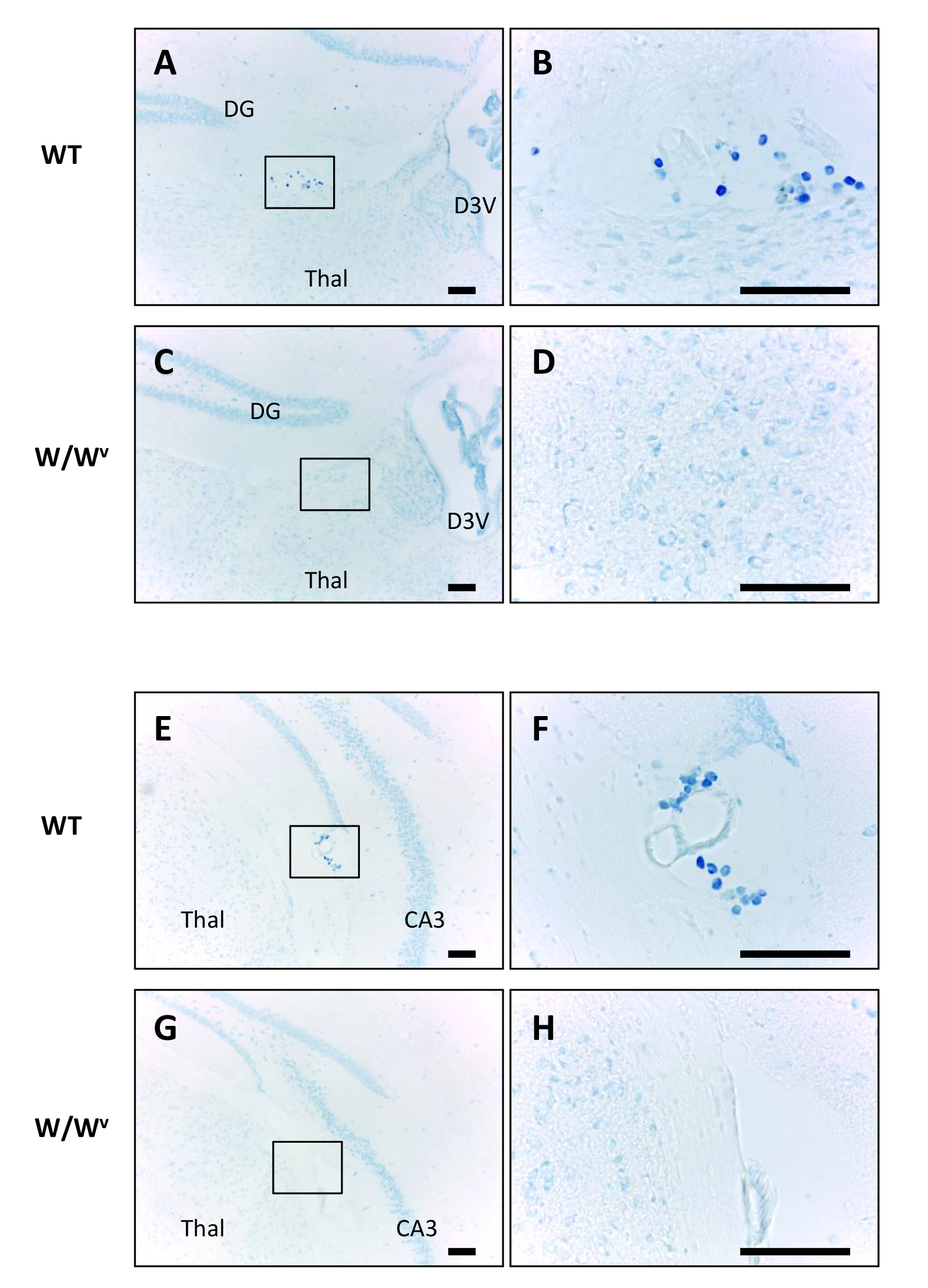

Supplement: Figure S1 — Connective tissue type mast cells in the brain of WT and W/Wv mice. Toluidine blue-stained mast cells using toluidine blue solution (0.05 %, pH2.5, Wako, Japan) are shown in the right side (A-D) and the left side (E-H) of the brain in WT (A, B, E and F) and W/Wv (C, D, G and H) mice. B, D, F and H show enlargements from the boxed regions at left (A, C, E and G) respectively. Scale bars indicate 100 μm. Abbreviations: dentate gyrus (DG), dorsal 3rd ventricle (D3V), thalamus (Thal), field CA3 hippocampus (CA3). (TIF) [file pone.0078434.s001.tif]

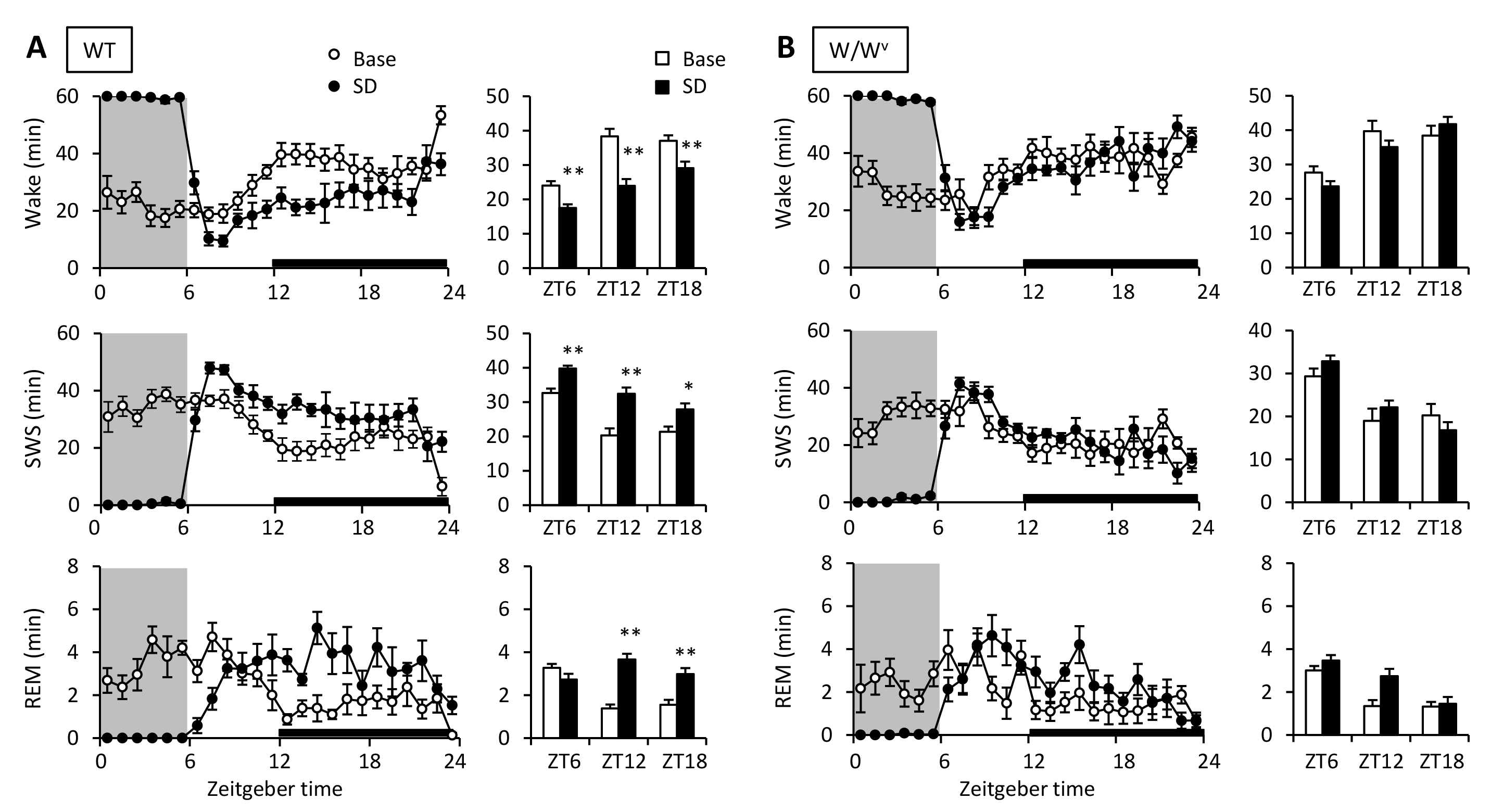

Supplement: Figure S2 — Rebound response after 6-hour sleep deprivation in (A) WT and (B) W/Wv mice. Time courses of sleep/wake for every hour during and after sleep deprivation are shown in the left panel. Sleep deprivation began at ZT0 and ended at ZT6 (the shadow areas). In the bar graphs (right panel), average amount of sleep/wake was calculated for each of the 6-hour periods across ZT6-12 (ZT6), ZT12-18 (ZT12), and ZT18-24 (ZT18). *p < 0.05, **p < 0.01, WT versus W/Wv mice. All data is expressed as mean ± SEM (n = 8/group). (TIF) [file pone.0078434.s002.tif]

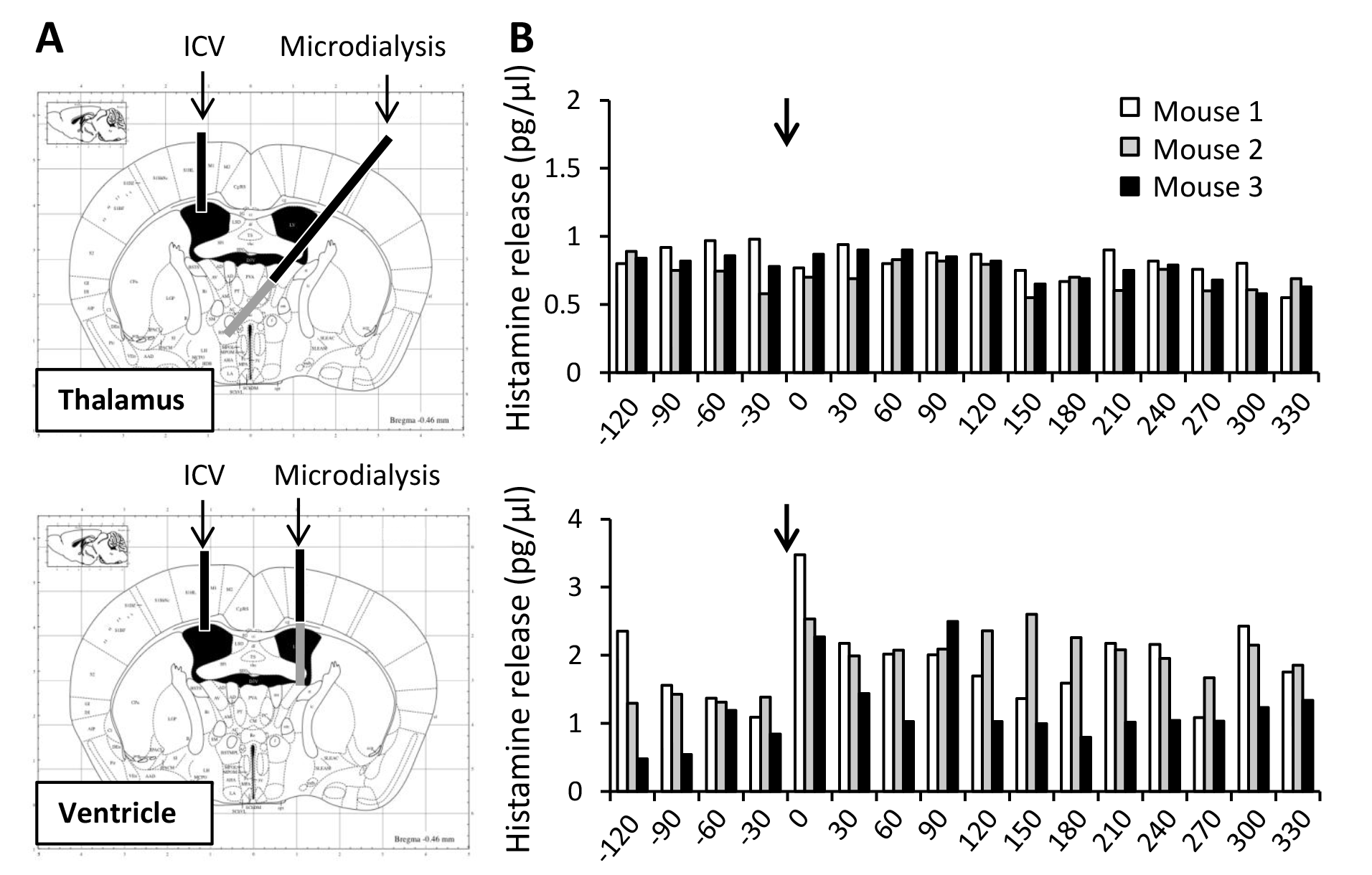

Supplement: Figure S3 — The effects of icv injection of compound 48/80 (C48/80) on histamine levels in WT mice. (A) A schematic representation of thalamus and lateral ventricle sections adopted from Franklin and Paxinos (Franklin and Paxinos, 2008). Black bars indicate the placement of the guide cannulae for icv injection of C48/80 and microdialysis probe. Microdialysis membranes, indicated as gray bars, were inserted into the thalamus (top panel) and lateral ventricle (bottom panel). (B) Extracellular histamine levels in the thalamus (top panel) and lateral ventricle (bottom panel) were measured by mast-cell stimulation of 5 μg C48/80 in each WT mouse (n=3). Each column represents the histamine levels for 30 minutes. The arrow (↓) indicates the time of the C48/80 injection. (TIF) [file pone.0078434.s003.tif]
